# Effects of Online Learning on College Students in Eastern China: A Structural Equation Model

**DOI:** 10.3389/fpubh.2022.853928

**Published:** 2022-03-17

**Authors:** Junqi Zhu, Haixia Zhao, Xue Wang, Li Yang, Zhiyuan Qin, Jichao Geng

**Affiliations:** ^1^School of Economics and Management, Anhui University of Science and Technology, Huainan, China; ^2^School of Earth and Environment, Anhui University of Science and Technology, Huainan, China

**Keywords:** learning behavior, learning cognition, learning effect, learning environment, online learning

## Abstract

With the spread of COVID-19 worldwide, online education is rapidly catching on, even in some underdeveloped countries and regions. Based on Bandura's ternary learning theory and literature review, this paper takes online learning of college students as the research object and conducts an empirical survey on 6,000 college students in East China. Based on literature review and factor analysis and structural equation model, this paper discusses the relationship among learning cognition, learning behavior, and learning environment in online learning of college students. The learning behavior includes interactive communication, self-discipline mechanism, classroom learning, and study after class. The learning environment includes teaching ability, knowledge system, platform support, process control, and result evaluation; learning cognition includes learning motivation, information perception, and adaptability. It is found that the learning environment has a significant positive impact on learning behavior, and learning cognition has a significant positive impact on learning behavior. It is uncertain whether the learning environment significantly impacts learning cognition. At the learning environment level, the teaching ability (0.59) has the most significant impact on the learning environment, followed by result evaluation (0.42), platform support (0.40), process control (0.33), and knowledge system (0.13). In terms of the influence on learning behavior, classroom learning has the most significant impact (0.79), followed by self-discipline mechanism (0.65), study after class (0.54), and interactive communication (0.44). In terms of learning cognition, information perception (0.62) has the most significant influence, followed by learning motivation (0.50) and adaptability (0.41). This paper suggests strengthening the construction of platforms and digital resources to create a more competitive learning environment. Improve process management and personalized online services, constantly stimulate students' enthusiasm for independent online learning, and cultivate students' online independent learning ability to promote the sustainable and healthy development of online education.

## Introduction

With the in-depth development of network technology, online teaching based on computer networks brings great advantages, such as resource diversification, implementation of openness, learning autonomy, and management automation (1). It also facilitates the implementation of life's fundamental tasks of cultivating people, promoting education and teaching reforms, and innovating talent (2). It is very important to cultivate and develop quality education and lifelong education, build a learning society, and achieve educational equity. The sudden outbreak of COVID-19 at the end of 2019 and the guiding principle of “stop classes, not schooling” have further accelerated the popularization and implementation of online education (3). Online education uses network and multimedia technology and is thus more flexible than traditional teaching methods (4). The effective use of a network teaching platform can compensate for the shortcomings of traditional classroom teaching models and improve teaching efficiency (5). However, because of the lack of supervision and necessary process assessment, the effects of online learning remain unclear (6, 7). On the basis of a literature review and field interviews, we summarize the current outstanding contradictions in online learning.

Online learning is a novelty for many students, who are faced with a vast array of online education resources from which to choose (8). Students obtain knowledge of these resources mostly through the recommendations of teachers and classmates or through instructions on the educational administration system platform when selecting courses (9). It is challenging to select courses without being familiar with the teaching content and teachers (10, 11). For important information such as teaching styles and effects, choices are made on the basis of the course name, course introduction, and/or other, intuitional information, which significantly increases the likelihood of blindly choosing courses (12). In addition, interviews with students have revealed that they do not really care whether the course is good, but whether it is easy to pass the assessment (13); the goal of online learning is thus limited to earning enough credits. Researching online courses and finding that there is insufficient information is likely to create a psychological gap (14). Unfortunately, online information is not positively focused on the curriculum, which further reduces learning choices (15).

The biggest obstacle to online education is the lack of face-to-face communication, especially the lack of body language interaction (16). Although online teaching platforms provide a wealth of learning resources and powerful interactive functions (17), in reality, most students go no further than the primary stage of browsing lecture videos, participating in required discussions, and completing their homework (18). Curriculum bulletin board system (BBS) forum are generally not active enough; the frequency of exchanges between teachers and students (and among students) is low, and the curriculum's social, pedagogical, and cognitive levels are not high (19). Online classes are divided into different modes: recording, broadcasting, and live broadcasting. In the recording and broadcasting teaching mode, teachers cannot immediately apprehend student dynamics and ease students' doubts. In the live teaching mode, because of the large number of students watching the live online class, it is difficult for lecturers to consider all the students during the interaction (20). Although online education platforms such as Erya provide a wealth of high-quality resources for college students, online interactive communication is generally completed by teaching assistants rather than by the lecturers themselves, which greatly reduces teacher–student communication (21). Students gave the following feedback: “The professors on the Erya General Studies course spoke well, but the content was too much, the class duration was long, and there was no communication. It was difficult to concentrate and listen throughout the period (22).” In other words, there were occasions when they wanted to listen, but they did not have the patience (23).

The modular operation of online platforms cannot meet the diversified learning needs of students. In interviews, students generally report that online courses are somewhat random and lack scientific guidance (24). The curriculum content setting is not very rational, some of it is too professional, much of it involves topical micro-courses, and the curriculum is small (25). If you want to learn a specific aspect in depth, you have to take multiple courses simultaneously and have an organized knowledge system. This undoubtedly reduces the learning enthusiasm of students from different majors (26). Because of the limited number of students allowed per class, many students miss out on the courses that interest them. Consequently, students who enroll too late have to choose less suitable courses to collect sufficient credits. Online learning is meaningful, but it is not a simple process (27).

A significant problem of online learning is that course assessment results do not truly reflect the degree of effort expended by students (28). The usual practice in online course assessments is for all the course videos to be played and the students to complete the homework requirements: that is, complete the course learning tasks. Students can cut and paste a large amount of work from the Internet to complete the final assessment (29). Another problem is that when the course administrator exports the list of students who submitted assignments from the teaching platform system, some may fail the course assessment because of technical issues. These simple assessment methods lack the process management of students' learning attitudes and learning styles and cannot control the phenomenon of “swiping lessons” (30). Quite a few students do things that have nothing to do with learning under the guise of online learning. Although they have opened the online learning homepage, they are actually randomly browsing the web. The administrator cannot be sure whether the student is learning (31). In addition, it is difficult to prevent plagiarism and cheating during online Internet tests (32). Although many online courses have been launched on platforms that have incorporated anti-cheating mechanisms, these technologies often lag behind the current level of network technology, and students can easily crack them through simple operations. As a result, the performance of students who are seriously concerned about online learning is not as good as that of students who are perfunctory, which in turn leads to the problem of “adverse selection” in online learning (33).

Owing to the above problems, we used Bandura's ternary learning theory and separated out the index system that influences the online learning process. We proposed corresponding research hypotheses, designed questionnaires, examined the factors and weights that influence students' online learning, and proposed feasible strategies to improve online learning effectiveness based on the weighted results.

## Literature Review

Bandura believes that the learning process comprises the three elements of learning cognition, learning behavior, and learning environment. It constitutes a learning system that has a profound impact on the learning effect (34). This also applies to online learning by college students. The online learning environment is shared, open, and efficient (35). It breaks the limitations of time and space and allows individual students to learn independently anytime and anywhere (36). The autonomous environment of the online learning process highlights the autonomy of students' learning behavior. Network technology provides students with the opportunity to actively participate in learning. At the same time, individual cognition has a guiding effect on behavior, and individual self-cognition stimulates and maintains the generation and development of behavior (37). Whether the learner is active in the online learning process determines the effectiveness of learning. Therefore, the key to clarifying the effect of online learning on college students is to accurately analyze and specify the relationship between online learning behavior, learning cognition, and learning environment (38). Previous literature studies on this aspect are as follows.

### Online Learning Behavior

The online learning behavior of college students can be observed through four indicators: interactive communication, self-discipline, classroom learning, and after-school learning (39–41). The interactive function of the network teaching platform is mainly embodied in human–computer interaction and human–human interaction. Human–computer interaction is achieved through the learner's browsing of platform texts, pictures, animations, videos, and taking quizes, developing models, etc. (42); human–human interaction is mainly achieved through interaction between learners and teachers and between learners (43). Through the course discussion module, teachers can answer queries from students according to the teaching content, and various forms of discussion can be carried out between students and groups to realize the benign interaction between teachers and students and between students (44, 45). Communication refers to the use of diversified methods in online learning communication management, with the help of new media tools such as network tools and mobile terminals for online and offline communication (46). The self-discipline mechanism is self-controlled in online learning. Online courses are highly flexible and require high levels of self-control (47). Unfortunately, some students, they concentrate on time and rush to complete the learning tasks, play the lecture videos without watching them, and use software to swipe lessons (48).

### Online Learning Cognition

College students' learning cognition in relation to online learning can be observed through three indicators: learning motivation, information perception, and adaptability (49–51). When choosing a subject to learn, college students do not passively accept environmental information but selectively adjust their attention on the basis of their current cognitive level, actively discovering and exploring the objective world (52). Motivations for online learning include satisfying curiosity, acquiring knowledge and skills, seeking personal challenges, seeking pleasure, supplementing professional learning content, communicating with others, improving education, and obtaining credits (53). Two of the above motivations (acquiring knowledge and skills and obtaining credits) have been highlighted (54); their frequency is also the highest. There are thus cases in which some students passively choose online learning to meet credit requirements. Information perception refers to students' reaction to information (55). That is, students can view course announcements, the syllabus, teaching plans, and other teaching materials on the network platform and can follow the requirements to study independently to meet course requirements. Adaptability refers to students' ability to adapt to online teaching, completing the role transition from “dependent learner” to “autonomous learner” (56).

### Online Learning Environment

The online learning environment for college students can be observed in terms of five indicators: teaching level, knowledge system, platform support, process control, and result evaluation (57–59). Teaching level refers to the ability of the teachers and the quality of the teaching. The term knowledge system refers to the quality of curriculum design, including whether the curriculum content system's division is reasonable, whether the media design is scientific, and whether the selection of knowledge points is complete (60). The term platform support refers to network assistance, helping students to learn online through course selection, tutoring and feedback, online testing, online examinations, etc. (61). The school can keep track of students' learning status at any time and oversee online management, online statistics, and other teaching activities (62). Process control can improve the learning efficiency of human–computer interaction (63). Result evaluation is an important way for teachers to measure the effect and quality of students' learning (64). It helps teachers understand the degree of proficiency in students' knowledge and facilitates students' cognition of self-learning (65). In the design and implementation of the entire online learning platform, scientific and effective learning evaluation can play a role in the supervision and guidance of students' online learning behavior (66).

On the basis of the foregoing, this study believes that the effect of college students' online learning is mainly affected by potential variables such as online learning behavior, online learning cognition and online learning environment, and proposes the following hypotheses (Table 1):

Hypothesis 1: The learning environment has a significant impact on online learning behavior.
Hypothesis 2: The online learning cognition has a significant impact on online learning behavior.
Hypothesis 3: The online learning environment has a significant impact on online learning cognition.

**Table 1:** The element system of the effect of online study on college students.

**Latent variable**	**Observed variable**	**Question**
Learning behavior	Self-discipline mechanism Q1	I was able to self-supervise and complete the learning while taking online courses
	Interactive communication Q2	I can participate in the communication during the online learning process
	Classroom learning Q3	I was able to pay attention during the online course
	Study after class Q4	I was able to complete the online exams and homework independently
Learning cognition	Learning motivation Q5	I take online courses to enrich myself
	Information perception Q6	I know the details of the online courses
	Adaptability Q7	I am comfortable with online courses
Learning environment	Teaching ability Q8	I recognize the quality of the online course teachers
	Knowledge system Q9	My online course has a complete knowledge system
	Platform support Q10	The online course learning process is not perfect network platform, often appear problems
	Process control Q11	Online course process supervision is of little use
	Result evaluation Q12	I have learned a lot from online courses

The innovation of this study lies in the identification of three potential variables (online learning behavior, online learning cognition and online learning environment) that affect the effect of college students' online learning by sorting previous literature. To clarify the interaction between potential variables, we constructed the evaluation model and paid attention to the path integration of the effect of college students' online learning and its influencing factors. Through the structural equation model (SEM), the weight of each factor between effects was analyzed and the derived factors that affect the effect of college students' online learning were explored. Later, feasible suggestions were put forward which were aimed to better promote the healthy development of network education.

## Research Design

### Sampling and Questionnaire Distribution

To verify the above hypotheses and explore the relationship between the latent and observed variables, this paper first conducted a face-to-face interview. Considering the subjective tendency in the interview, in order to avoid the occurrence of this problem, we have drawn up an interview outline in advance. By summarizing the 142 interviews results and further reviewing the literature, this paper discusses the relationship among learning cognition, learning behavior, and learning environment in online learning of college students. The learning behavior includes interactive communication, self-discipline mechanism, classroom learning, and study after class. The learning environment includes teaching ability, knowledge system, platform support, process control, and result evaluation; learning cognition includes learning motivation, information perception, and adaptability. In addition to the four basic information items, the 12 observed variables of the formal questionnaire use a 7-point Likert scale to measure the respondents' different attitudes or tendencies. The grading level comprised complete disagreement (1 point), comparatively inconsistent (2 points), inconsistent (3 points), uncertain (4 points), basically in line (5 points), relatively in line (6 points), and completely in line (7 points). See Table 1 for further details.

This study used a combination of non-probability and simple random sampling.

Step 1, 12 prefecture-level cities from six provinces and one city in East China were randomly selected as primary sampling units.
Step 2, 12 colleges and 16 universities from the 12 prefecture-level cities were selected as secondary sampling units.
Step 3, Each grade from 12 colleges and 16 universities were used as the third unit.
Step 4, Students in each class were randomly selected as the final sampling unit.

According to the calculation formula of sample size *N* = *Z*^2*^[P^*^(1 – *p*)/*E*^2^], we set the confidence level to 95% (*Z* = 1.96), error value *E* = 5%, probability value *P* = 0.5, and calculate the sample size *N* = 384, so our sample size should be above 384. In order to ensure the comprehensiveness, scientificity and representativeness of the survey, and in consideration of the fact that there may be invalid questionnaires in the actual survey, we distributed 500 questionnaires (500 > 384) in each 12 prefecture-level primary sampling units, and a total of 6,000 college students were distributed.

The survey took the form of verbal inquiries and self-administered questionnaires, which were collected by the investigators on the spot. During this process, the research team strictly controlled the selection of investigators, supervision of quality, and management during the survey implementation process to ensure the accuracy and completeness of the questionnaire. Through a manual review of the questionnaire data, missing and invalid questionnaires were eliminated; 4,861 questionnaires were finally determined to be valid samples, and the questionnaire's effective response rate was 81%.

### Demographic Characteristics of Survey Samples

The sample's demographic characteristics mainly included basic information, such as gender, grade, education level, and students' majors (Table 2). The proportions of men and women in the survey sample were 46 and 54%, respectively. The proportions of first-year students, sophomores, juniors, and seniors were 10.3, 48, 38.5, and 2.2, respectively. In terms of majors, science and engineering students accounted for 51% of the overall sample, literature and history students accounted for 39%, and agricultural and medical students accounted for 10%.

**Table 2:** Participants' demographic characteristics.

**Variable**	**Variable definitions**	**Percentage (%)**	**Variable**	**Variable definitions**	**Percentage (%)**
Gender	Male	46	Grade	Freshman	10.3
	Female	54		Sophomore	48
Major	Literature and history	39		Junior	38.5
	Science and technology	51		Senior Year	2.2
	Agricultural medicine	10	Education level	Undergraduate	100

### Questionnaire Analysis

SPSS software (version 26.0) was used for statistical analysis. First, participants' total scores were sorted in ascending order, and the top 3% of the samples were classified as low groups; the 3% after the scores were classified as high groups, and then extreme value comparisons were made.

Then, an independent sample *t*-test was performed, and the sig value of the mean equation was found to be <0.005, indicating that there were significant differences between the low and high groupings in all variables, which is in line with the actual situation. On this basis, we performed the same qualitative inspection for each item.

#### Reliability Test

In this study, the internal consistency method was used to test the reliability of the variables, and the reliability of the questionnaire items was analyzed. The α reliability coefficient of the measurement variable was calculated to be 0.872, which is an acceptable value, >0.7, indicating the very high reliability of the scale.

#### Validity Test

Validity is the degree to which the measurement results reflect the content to be examined; it can be divided into content and construct validity. To ensure that the scale met content and validity requirements, we referred to a large number of survey theories and literature during the design of the online learning effect questionnaire and consulted a professional instructor. They agreed that the questionnaire and indicator collection points were essentially the same. In this study, the factor analysis method was used to analyze the scale construction's validity using the Kaiser–Meyer–Olkin (KMO) and Bartlett sphericity tests. The results show that the KMO value of the scale is 0.895 (>0.8), which is close to 1, indicating that the sample size meets the requirements and that the data are suitable for factor analysis, while the significance level of Bartlett's sphericity test is *p* = 0.000 <0.01, indicating that the original variables have a meaningful relationship, and the scale data are suitable for factor analysis. In summary, this questionnaire has good reliability and validity and can effectively investigate the effects of online learning on college students.

#### The Difference Analysis of Sociodemographic Variables

The difference analysis mainly focuses on demographic variables and studies the influence of college students' gender, grade, major, education level, and other factors on online learning behavior. Independent sample *T*-test and one-way ANOVA were used for variables using SPSS 26.0 software. Independent sample *T*-test was used for the influence of gender, and single-factor ANOVA was used for the impact of grade and major on learning behavior. Since all the subjects in this survey are undergraduates, there is no difference in the influence of their education level on online learning behavior.

(1) Gender. An independent sample *T*-test was used to analyze the influence of gender in this paper, and the analysis results are shown in Table 3. It can be seen from Table 3 that the significance level of the gender of college students on online learning behavior is 0.738, much higher than 0.05. The null hypothesis of the independent sample *T*-test is accepted, which means there is no significant difference between the gender of college students in learning behavior.

**Table 3:** The independent sample *T*-test of gender online learning behavior.

**Variable**	**Male**	**Female**	**T**	**P**
Learning behavior	6.2384 ± 0.79233	6.3000 ± 0.49070	−0.335	0.738

(2) Grade. This paper adopts one-way ANOVA to analyze the influence of college students' grades on online learning behavior, and the analysis results are shown in Table 4. According to one-way ANOVA in Table 4, college students of different grades have significant differences in online learning behaviors. After the LSD *post-test*, the mean value of junior grade was 4.4583, higher than that of other grades. The performance of junior students in online learning behavior is better than that of other grades.

**Table 4:** ANOVA test of grade in online learning behavior.

**Grade**	**Online learning behavior**	
	**Mean**	**Standard deviation**
Freshman	6.3015	0.68868
Sophomore	6.1429	0.85394
Junior	6.4583	0.45871
Senior year	6.4286	0.55367
*F*	2.142*	
LSD postmortem test	3 > 1, 2, 4	

Note: *P < 0.1, **P < 0.05, ***P < 0.01.

(3) Major. This paper adopts one-way ANOVA to analyze the influence of college students' majors on online learning behavior, and the analysis results are shown in Table 5. As shown in Table 5, the significance was 0.929 > 0.05, indicating that college students' majors in this study have no significant difference in online learning behavior.

**Table 5:** ANOVA test of different majors in Online Learning of college students.

**Majors**	**Onlin learing behavior**	
	**Mean**	**Standard deviation**
Literature and history	6.1994	0.80402
Science and technology	6.3424	0.64412
Agricultural medicine	6.2480	1.01036
*F*	0.929	

#### Principal Component Analysis

Principal component analysis was used to extract factors with feature values >1 to achieve clustering of various online learning effects on college students. SPSS software extracted three common factors with a cumulative variance contribution rate of 66.092%. Thus, it can be said that the three extracted common factors can effectively explain elements of the online learning effect. Third, to further test each element of the online learning effect on college students, the reliability of the three extracted common factors was tested. The results showed that each element's item-total correlation coefficients were all higher than 0.5, and the Cronbach's α coefficients of each factor were all between 0.811 and 0.920, in line with research standards. The three principal component factors were learning behavior, learning cognition, and learning environment. The output rotated component matrix and the names of the factors are listed in Table 6.

**Table 6:** Component matrix after rotation.

**Online learning effect factor (dimension)**	**Element**	**Ingredient**		
		**1**	**2**	**3**
Learning behavior	Q1	0.733	0.359	0.119
	Q2	0.549	0.382	0.013
	Q3	0.552	0.231	0.127
	Q4	0.640	0.096	0.353
Learning cognition	Q5	0.216	0.593	0.395
	Q6	0.321	0.628	0.202
	Q7	0.274	0.511	0.041
Learning environment	Q8	0.327	0.129	0.523
	Q9	0.306	0.271	0.509
	Q10	0.409	0.492	0.558
	Q11	0.206	0.179	0.550
	Q12	0.445	0.066	0.603
Eigenvalues		2.890	1.686	1.033
Percentage of variance		30.897	19.864	15.331
Cumulative percentage		30.897	50.761	66.092

## Model Construction

### Measurement Model

After standardizing the basic data of the three latent variables and 12 observed variables, we introduced them into the evaluation system. The confirmatory factor analysis (CAF) method can be used to obtain the three principal component factors of the 12 observed variables on the three latent variables; the variance of the three principal component factors was fixed at 1 (fixed coefficient). The measured variable of each latent variable was no longer defined as a reference index variable.

**Figure 1:**
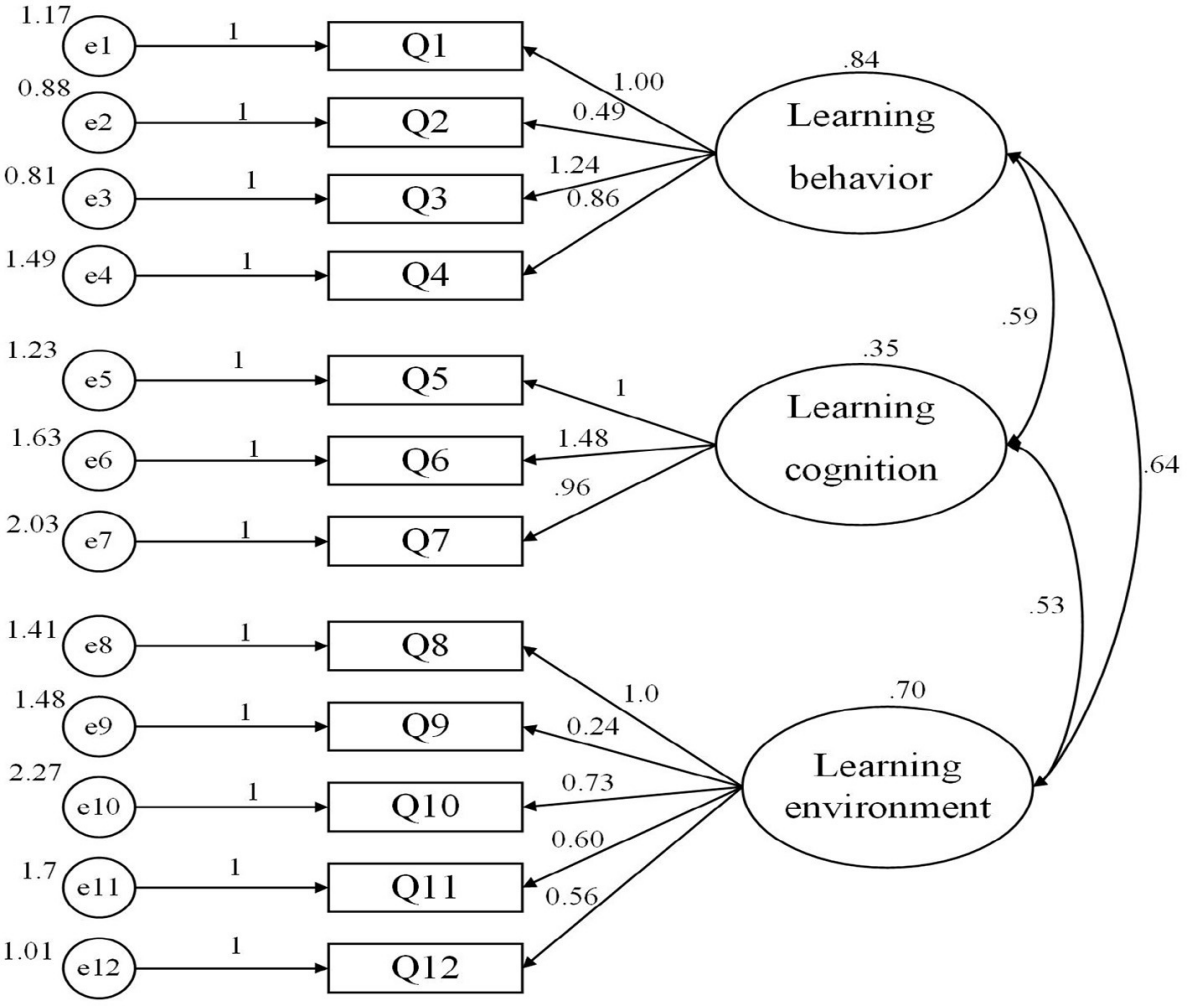
Structure diagram of the measurement model of online learning's effect on college students.

The model calculation results show that the fitting results of the online learning effect measurement model for college students (Figure 1) are ideal. The CAF measurement model path diagram estimation results are as follows: the model can be identified and converged, and the non-standardized estimated value model has no negative error variance as a whole. If the absolute value of the standardized path coefficient is greater than or close to 1, the model does not violate the model identification rules. The overall model fit chi-square value/degree of freedom value of 2.735 (<3), goodness of fit index (GFI = 0.954), comparative fit index (CFI = 0.915), and adjusted goodness of fit index (AGFI=0.922) were above the standard or critical value of the fit (0.9), and root mean square error of approximation (RMSEA = 0.068) was lower than the standard value of 0.08. All statistical indicators are in line with the standard (Table 7), and the fitting effect of the measurement model was relatively satisfactory.

**Table 7:** Tests on the factors of measurement model fit.

**Statistical test volume**	**Adapted standard or critical value**	**Measurement model**
χ^2^	*P* > 0.05 (Not significant)	87.528 (*p =* 0.000 <0.05)
Chi-square degree of freedom ratio	<3.00	2.735
RMSEA (root mean square error of approximation)	<0.08	0.068
GFI (goodness of fit index)	>0.90	0.954
AGFI (adjusted goodness of fit index)	>0.90	0.922
CFI (comparative fit index)	>0.90	0.915
PNFI (parsimonious normed fit index)	>0.50	0.622
PGFI (parsimonious goodness-of-fit index)	>0.50	0.555

### Structural Model

#### Hypothetical Model

**Figure 2:**
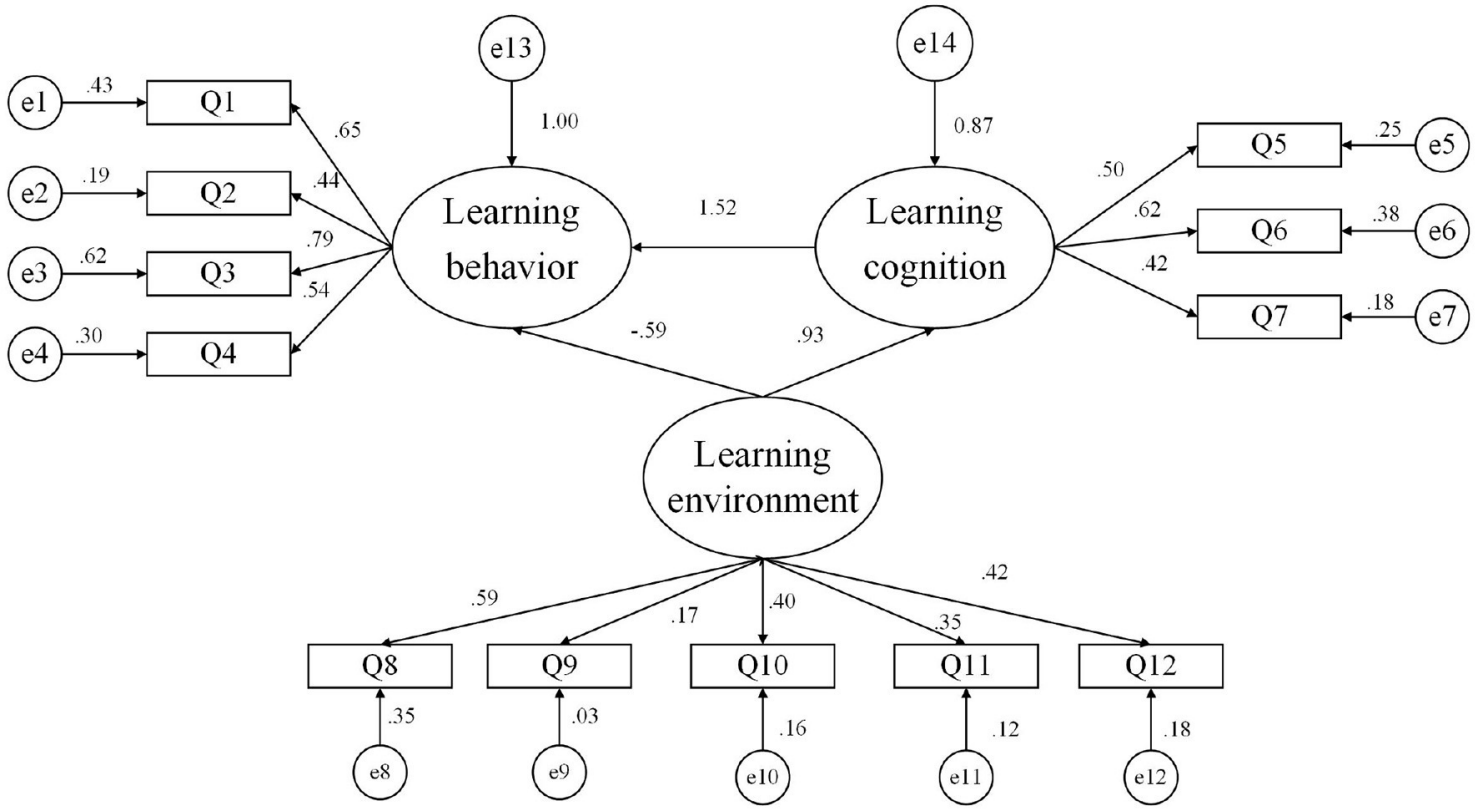
Initial structure diagram of the measurement model of online learning's effect on college students.

Following the theoretical model, SEM (Structural Equation Model) is used to study the main structure of online learning behavior, learning cognition, and the learning environment. Individual learning behavior was the dependent variable, learning cognition was the mediating variable, and learning environment was the independent variable; learning environment uses learning cognition as the mediator to act on learning behavior. On the basis of these assumptions, three initial hypothesis model structural equations of latent variables were established (Figure 2).

The model fit results (Table 8) show that the fit chi-square value/degree of freedom value = 2.735, RMSEA = 0.068, GFI = 0.954, AGFI = 0.922, CFI = 0.915, parsimonious normed fit index (PNFI) = 0.622, and parsimonious goodness-of-fit index (PGFI) = 0.555, and the index data met the parameter requirements. However, norm fit index (NFI) = 0.874 and tucker-lewis index (TLI) = 0.880, which are <0.9, did not meet the parameter requirements. The model fits well but needs to be revised.

**Table 8:** Results of the measurement model fitness test.

**Statistical test volume**	**Adapted standard or critical value**	**Measurement model**
χ^2^	*p >* 0.05 (Not significant)	87.528 (*p =* 0.000 <0.05)
Chi-square degree of freedom ratio	<3.00	2.735
RMSEA (root mean square error of approximation)	<0.08	0.068
GFI (goodness of fit index)	>0.90	0.954
AGFI (adjusted goodness of fit index)	>0.90	0.922
CFI (comparative fit index)	>0.90	0.915
PNFI (parsimonious normed fit index)	>0.50	0.622
PGFI (parsimonious goodness-of-fit index)	>0.50	0.555
NFI (norm fit index)	>0.90	0.874
TLI (Tucker-Lewis index)	>0.90	0.880

#### Model Modification

**Figure 3:**
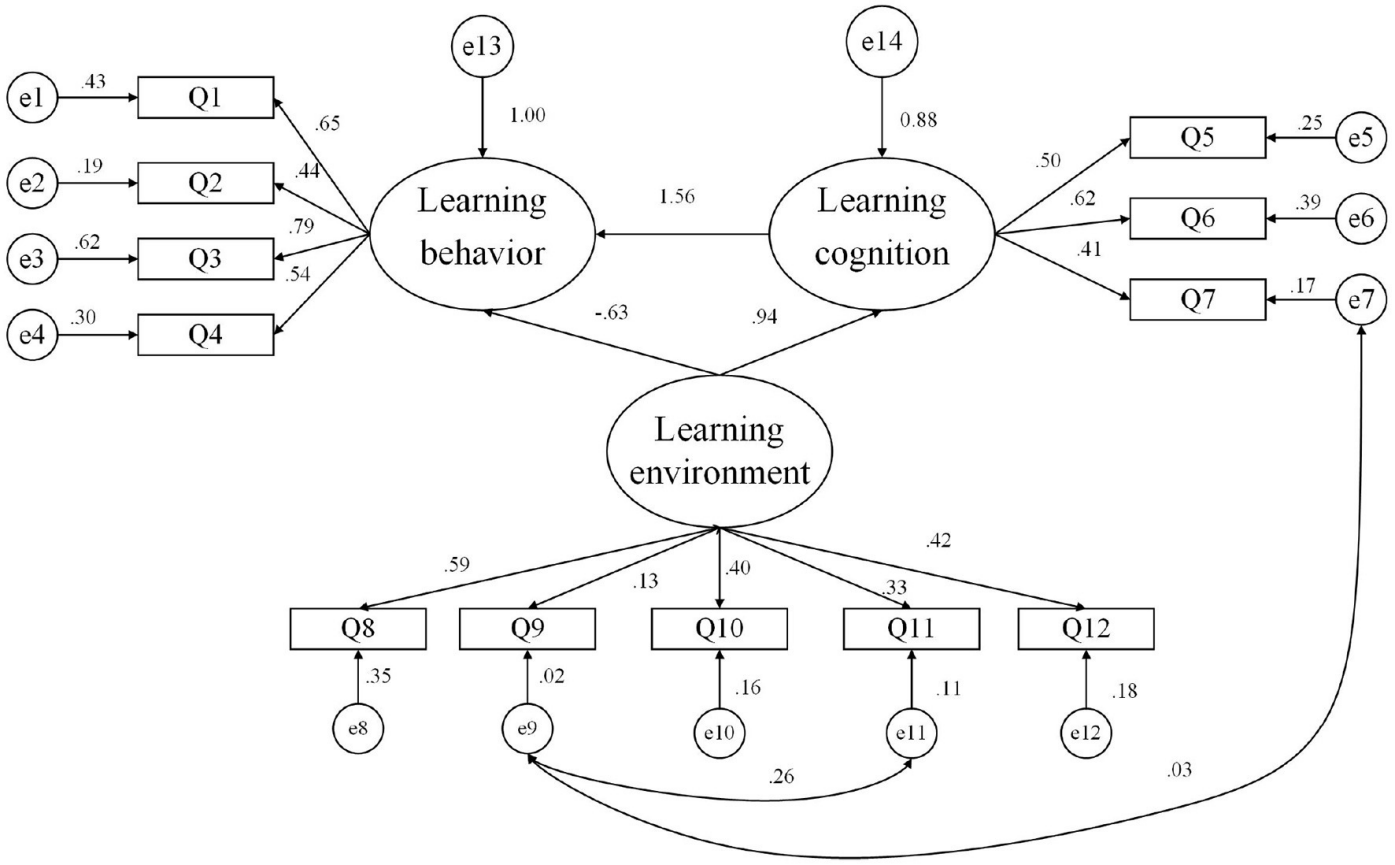
The revised structural equation model.

In this study, increasing collinearity with other indicator residuals was used to improve the degree of fit, observe the Modification Indices (MI) value of the output result, and increase the covariance between the two residuals with a larger MI value. The MI values of e7 and e11 were 44.959, which exceeded the other values. This proves that if the correlation path between the explicit variables Q7 and Q11 is increased, the chi-square value of the model decreases by 44.957. From a theoretical analysis, the stronger the student's “adaptability,” the less effort the “process control” will require. The two are related, so it is feasible to increase the residuals' relative paths, e7 and e11. Similarly, the MI values of e9 and e11 were 24.876, which exceeded the other values. If the correlation path between the explicit variables Q9 and Q11 increases, the model's chi-square value decreases by 24.876. From a theoretical analysis, the “system of knowledge” affects “process control,” which will, in turn, further affect the “knowledge system.” The two are related, so it is also feasible to increase the relative path of residuals e9 and e11. The revised structural equation model is shown in Figure 3.

#### Model Checking

First, before the fit test of the comprehensive evaluation model, the model must be tested for “violation of estimates” to check whether the model fit coefficient exceeds the acceptable range. Generally, there are two test items for “violation estimation”: whether there is a negative error variance and whether the standardized coefficient exceeds or is too close to 1. On testing (Table 9), no negative error variance was noted in the model; the absolute value of the model's standardized coefficient was 0.13–0.85, and none of them exceeded 0.95. Therefore, the model did not have irregular estimates, and the overall model fit could be tested.

**Table 9:** Model fitness test results after modification.

**Statistical test volume**	**Adapted standard or critical value**	**Measurement model**
χ^2^	*p >* 0.05 (Not significant)	56.419 (*p =* 0.003 <0.05)
Chi-square degree of freedom ratio	<3.00	1.820
RMSEA (root mean square error of approximation)	<0.08	0.047
GFI (goodness of fit index)	>0.90	0.970
AGFI (adjusted goodness of fit index)	>0.90	0.947
CFI (comparative fit index)	>0.90	0.961
PNFI (parsimonious normed fit index)	>0.50	0.633
PGFI (parsimonious goodness-of-fit index)	>0.50	0.547
NFI (norm fit index)	>0.90	0.919
TLI (Tucker-Lewis index)	>0.90	0.943

Second, in terms of model fit evaluation, the higher the model fit, the higher the model availability and the more meaningful the parameter estimation. Chi-square statistics are often used to test the model fit. When the model fits the data, the difference is 0. The chi-square value of this model was 56.419, and the return probability was 0.003. Chi-square statistics are easily affected by sample size, so in addition to chi-square statistics, other fitness indicators need to be referred to at the same time. According to the data in Table 9, each statistic's indicators were up to standard, the fitting effect of the model was relatively satisfactory, and further research could be conducted.

#### Model Results

In the online learning effect model for college students, the path-effect relationship between independent, intermediate, and dependent variables is shown in Table 10. The analysis results show that the learning environment's path acts on learning cognition, and that of learning cognition acts on learning behavior. The construct reliability (CR) value met the requirement (>1.96); the standardized estimated value was significant, and all were positive. The CR value of the learning environment acting on learning behavior was <1.96, which was not significant. Therefore, Hypothesis 1 (the learning environment has a significant positive impact on learning behavior) and Hypothesis 2 (learning cognition has a significant positive impact on learning behavior) are both valid, and Hypothesis 3 (the learning environment has a significant positive impact on learning cognition) requires further exploration.

**Table 10:** Path-effect relationship between independent, intermediate, and dependent variables.

**Path**	**Estimate**	**S.E**.	**CR**	**P**
Learning cognition ← Learning environment	0.369	0.075	4.928	***
Learning behavior ← Learning environment	−0.078	0.050	−1.554	0.120
Learning behavior ← Learning cognition	0.786	0.115	6.819	***

Note: *P <0.1, **P <0.05, ***P <0.01.

## Conclusion and Discussion

### Conclusion

The study found that the online learning environment has a significant positive impact on learning behavior, learning cognition has a significant positive impact on learning behavior, and whether the learning environment significantly affects learning cognition is unclear. At the specific level, teaching level (0.59) had the greatest impact on the learning environment, followed by result evaluation (0.42), platform support (0.40), process evaluation (0.33), and knowledge system (0.13). With regard to the impact on learning behavior, classroom learning (0.79) had the largest impact, followed by self-discipline (0.65), after-school learning (0.54), and interactive communication (0.44). With regard to the impact on learning cognition, information perception (0.62) had the greatest impact, followed by learning motivation (0.50) and adaptability (0.41).

### Discussion

On the basis of the above conclusions, we believe that it is necessary to consider the following factors to stimulate students' learning behavior and effectively improve college students' online learning.

#### Strengthening of Platform Support, Sharing of Digital Resources, and Cultivation of Students' Independent Learning Ability

With regard to the observed variables of the learning environment, given the importance of teachers' teaching level (0.59) and platform support (0.40), we believe that online platforms should provide students with massive co-construction and shared digital resource libraries to meet their independent learning needs [67]. From the perspective of resource sharing, the platform interface should provide convenient links so that students can directly view the learning courseware, and there should be no restrictions or problems that could hamper the generation of links (22). From the perspective of the timeliness of resources, the curriculum library should keep pace with the times, update the teaching content in a timely manner, and ensure that the online content is innovative and forward-looking. From the point of view of the pertinence of resources, the construction of an online information resource database should incorporate students' needs and the current social situation to reflect the characteristics of the curriculum (9).

#### Introduction of a Multiple Scoring Mechanism and a Medal Ranking System to Create a Competitive Learning Environment

In addition to the teaching level, result evaluation (0.42) has a higher impact on the learning environment, indicating that students value academic performance. Here, we recommend diversified evaluations of students' final scores. The establishment of a network medal system is also recommended, with the stipulation that the medal can be exchanged for learning scores, whether students learn course knowledge in the learning platform, answer classmates' doubts, share their homework, or publish themselves. Different types of medal can be obtained for their learning experiences or for comments on others' speeches. The medal level is evaluated by the degree of participation and speech quality (the number of likes and comments received by others). The higher the medal level, the higher the redeemable learning score counted in the final learning score. The network medal is a recognition of students' knowledge and ability and an award for their contributions to the platform's construction. Regular selection of outstanding people for material or spiritual rewards may also stimulate the sense of competition and enthusiasm to participate in constructing the sharing platform. A competitive learning environment can enable students to create a learning atmosphere of “you catch up with me” in online learning and ultimately achieve good learning outcomes.

#### Strengthening of Classroom Control, Information Output, Course Management, and System Maintenance

Classroom learning (0.79) is a key element that affects the learning environment and is a necessary condition for achieving learning self-discipline (0.65), which affects learning behavior. The management should strengthen the review of courses, ensure the quality of courses, set up online reporting stations, and adopt severe measures against speculative behaviors such as cheating in the learning process. Simultaneously, online courses should set up administrators to effectively test students' open learning, score discussion posts, and promptly block and delete posts with no substantive content to sanitize the learning environment and improve course management efficiency. Online course management departments should increase monitoring procedures, invest more in research and development, establish discussion and open work areas, and stipulate a minimum number of individual student posts. Moreover, they should add functions to automatically clean up blank, sign-in, messy, and other invalid posts to better maintain the system. Output information and course management should be combined effectively, and process control should be strengthened.

#### Personalized Pre-tests, Broadening of Channels of Information Perception, and Enrichment of the Learning Subject's Curriculum Cognition

Survey statistics show that only 5% of students believe that they fully understand the selected online course's basic information when choosing a course. This information perception significantly affects learning cognition (0.62), which shows that necessary pre-class guidance and course descriptions are very important. It is necessary to establish a pre-school test project, conduct personalized pre-tests for online course selection, equip corresponding online courses according to the students' interests, provide necessary online knowledge analysis, and arrange assignments during the learning process. In addition, before the start of online courses, the management department should adopt a combination of online and offline methods and make full use of the student union, clubs, the Academic Affairs Office's official website, the official WeChat, and school promotion windows to help students understand the types, contents, and basics of the online courses: that is, basic information regarding courses, relevant course evaluation, course selection time, and course selection method (60). Considering the factors of learning motivation (0.50) and adaptability (0.41), there is a need to establish a more detailed course classification system that clearly distinguishes professional and general courses, indicates the difficulty of the courses, and enables students to choose courses of interest on time, as well as courses that match their own abilities.

#### Addition of Discussion Classes, Provision of Real-Time Voice Answering Support, and Stimulation of Learners' Enthusiasm to Learn Independently

Given the significant impact of post-class communication on learning behavior, it is necessary to increase communication and interaction between teachers and students, as well as between the students themselves (41). In the online learning process, the barrage function and online Q&A should be enhanced. Students should be encouraged to ask questions and provide answers through the barrage function. In addition, a few trending topics should be selected every week according to the number of likes, as “hot posts,” and addressed by the lecturer and published online (9). When self-learning online, it is necessary to intersperse learning with course discussions and mid-term interactive discussions to understand learning progress. This promotes students' understanding of online learning content, expands their ways of thinking, enriches their imaginations, and teaches them self-control. Students also offer some kind of supervision. The discussion sessions used real-time multimedia interactive systems to support real-time Q&A and online interactions. An appropriate enhancement of the voice-linking function would facilitate interactive online communication (21).

## Contributions and Limitations

Using questionnaires and interviews, combined with factor analysis and structural equation modeling, this study examined the effects of online learning on college students and explored the relationship between college students' online learning and learning cognition, learning behavior, and learning environment. A significant relationship between the online learning environment, learning cognition, and learning behavior was discovered, and relevant suggestions were put forward to stimulate college students' learning behavior and improve their online learning abilities. However, this study was mainly carried out in East China; it is therefore necessary to further expand the scope of the research in follow-up studies and to add research data from other countries or regions to further verify and improve the results of this research.

## Data Availability Statement

The original contributions presented in the study are included in the article/supplementary material, further inquiries can be directed to the corresponding author/s.

## Author Contributions

JZ contributed to the analysis and interpretation of data for the study. HZ wrote the first draft of the manuscript. LY and XW designed the framework for this study. ZQ and JG contributed to the acquisition of data for this study. All authors have approved the final manuscript.

## Funding

This study was supported by the Key Teaching and Research Project of Anhui Province (2020jyxm0453), the Anhui Province Teaching Demonstration Class Project (2020SJJXSFK0832 and 2020SJJXSFK0918), the Key Teaching Research Project of Anhui University of Science and Technology (2021xjjy03), the Major of National Social Science Foundation of China (20ZDA084), and the Program of Humanities and Social Sciences in Colleges and Universities of Anhui Province (Nos. SK2020A0209 and SK2020A0210).

## Conflict of Interest

The authors declare that the research was conducted in the absence of any commercial or financial relationships that could be construed as a potential conflict of interest.

## Publisher's Note

All claims expressed in this article are solely those of the authors and do not necessarily represent those of their affiliated organizations, or those of the publisher, the editors and the reviewers. Any product that may be evaluated in this article, or claim that may be made by its manufacturer, is not guaranteed or endorsed by the publisher.
